# High-Order Correlation Integration for Single-Cell or Bulk RNA-seq Data Analysis

**DOI:** 10.3389/fgene.2019.00371

**Published:** 2019-04-26

**Authors:** Hui Tang, Tao Zeng, Luonan Chen

**Affiliations:** ^1^Key Laboratory of Systems Biology, CAS Center for Excellence in Molecular Cell Science, Institute of Biochemistry and Cell Biology, Shanghai Institutes for Biological Sciences, Chinese Academy of Sciences, University of Chinese Academy of Sciences, Shanghai, China; ^2^CAS Center for Excellence in Animal Evolution and Genetics, Chinese Academy of Sciences, Kunming, China; ^3^School of Life Science and Technology, ShanghaiTech University, Shanghai, China; ^4^Shanghai Research Center for Brain Science and Brain-Inspired Intelligence, Shanghai, China

**Keywords:** high–order, integration, clustering, single-cell, bulk data analysis

## Abstract

Quantifying or labeling the sample type with high quality is a challenging task, which is a key step for understanding complex diseases. Reducing noise pollution to data and ensuring the extracted intrinsic patterns in concordance with the primary data structure are important in sample clustering and classification. Here we propose an effective data integration framework named as HCI (High-order Correlation Integration), which takes an advantage of high-order correlation matrix incorporated with pattern fusion analysis (PFA), to realize high-dimensional data feature extraction. On the one hand, the high-order Pearson's correlation coefficient can highlight the latent patterns underlying noisy input datasets and thus improve the accuracy and robustness of the algorithms currently available for sample clustering. On the other hand, the PFA can identify intrinsic sample patterns efficiently from different input matrices by optimally adjusting the signal effects. To validate the effectiveness of our new method, we firstly applied HCI on four single-cell RNA-seq datasets to distinguish the cell types, and we found that HCI is capable of identifying the prior-known cell types of single-cell samples from scRNA-seq data with higher accuracy and robustness than other methods under different conditions. Secondly, we also integrated heterogonous omics data from TCGA datasets and GEO datasets including bulk RNA-seq data, which outperformed the other methods at identifying distinct cancer subtypes. Within an additional case study, we also constructed the mRNA-miRNA regulatory network of colorectal cancer based on the feature weight estimated from HCI, where the differentially expressed mRNAs and miRNAs were significantly enriched in well-known functional sets of colorectal cancer, such as KEGG pathways and IPA disease annotations. All these results supported that HCI has extensive flexibility and applicability on sample clustering with different types and organizations of RNA-seq data.

## Introduction

Cells, the fundamental unit in biology, can be distinguished by their size and shape using a microscope. Later, advanced technological developments have made it possible to isolate a large number of cells, and along with improvements in RNA isolation and amplification methods, next-generation sequencing technologies are used to profile the transcriptome of individual cells. Single-cell RNA sequencing (scRNA-seq) now allows for omics analysis of individual cells, which can expose exciting biological processes, novel medical insights and efficient clinical applications (Dunham et al., 2012; Kolodziejczyk et al., 2015; Wagner et al., 2016). The advances in single-cell technologies have led to more comprehensive studies for multicellular organisms than previous approaches. Recently, 10X Genomics could release a single-cell dataset of more than 1.3 million cells (2017)^1^. With the production of large amount of single-cell data, understanding the development of an organic organ requires to characterize all of its cell types, so that, it is important to quantify single-cell cell types with high quality. Conventionally, one key application of scRNA-seq is to cluster cell types based on cells' transcriptome profiles through unsupervised computational methods (Lloyd, 1982; Jaitin et al., 2014; Mahata et al., 2014; Grün et al., 2015; Kiselev et al., 2017; Jiang et al., 2018; Shi et al., 2018; Dai et al., 2019). These approaches in recently published studies show some good performances in determining different cell types (Xue et al., 2013; Patel et al., 2014; Pollen et al., 2014; Shalek et al., 2014). SAFE-clustering (Yang Y. et al., 2018) can take as input results from multiple clustering methods and scmap (Kiselev et al., 2018) can compare clusters across data sets without merging. RaceID (Grün et al., 2015) augments k-means to identify rare cell types by detecting outliers, but k-means faces the problem of global solution. Meanwhile, SC3 (Kiselev et al., 2017) adopts repeated application of *k*-means using a small subset of principal components or different initial conditions and finding the consensus clusters. SC3 is a user-friendly clustering method that works well for smaller datasets. However, it takes too long in terms of computation time because of amount of calculating correlation matrix of cells. Besides, CIDR (Lin et al., 2017) adapts hierarchical clustering (HCA) for single-cell datasets by adding an implicit imputation of zeros into the distance calculation. But, an important shortcoming of hierarchical clustering is that it is prohibitively expensive for large datasets. Therefore, the more efficient and accurate method is still urgently needed to cluster cell types.

At the same time, large amounts of bulk data have already become widely available resources along with rapid development of high throughput technologies. To take full advantage of these rich data sets, integrating multiple datasets will give more opportunities to address biological dynamics and cancer heterogeneity (Hamid et al., 2009; Wang et al., 2014). Some integration methods have been developed in recent years, such as: iClusters, SNF, NMF, and PFA (Zhang et al., 2011; Mo et al., 2013; Mahata et al., 2014; Wang et al., 2014; Shi et al., 2017). However, there are still several limitations of these approaches. For example, iClusterPlus is based on Gaussian assumption, which could not make sense when data is too heterogeneous on signal distributions. And recently developed pattern fusion analysis (PFA) can integrate multidimensional data (Shi et al., 2017) so as to provide a comprehensive way to understand biological processes and complex diseases in a multi-view manner. In theory, PFA can align local sample-patterns derived from each single data type into a global sample-pattern to characterize the sample types in a low-dimensional feature space, so that, it is expected that PFA can model the sample types (i.e., cell types) when using scRNA-seq. However, the original PFA is designed for multi-source data rather than only one source data, in addition to insufficient analysis on the sample features. Thus, it is required to extend the original PFA to sample clustering even for one source data by a unified integration framework.

To overcome above challenges, we proposed a unified computational framework for distinguishing single-cell cell types from single-cell RNA-seq data, which also keeps the ability for clustering sample types from bulk RNA-seq data. The new method named as HCI (High-order Correlation Integration), can integrate joint high-order correlation matrices, where the iterative use of Pearson's correlation coefficient in sample data are incorporated into our previously developed pattern fusion analysis method (PFA) (Shi et al., 2017). Technically, HCI integrates single-cell data sets and different distance matrices corresponding to different sample correlation feature spaces (i.e., the distance between the cells) by joint matrix factorizations.

On the one hand, HCI has been compared with other existing methods [i.e., SC3 (Kiselev et al., 2017) and SEURAT (Macosko et al., 2015)] for identifying cell types on various single-cell RNA-seq data. And the robustness of HCI was also tested in different correlation orders (e.g., one-order, second-order, different percentage of differentially expressed genes). Furthermore, a case study was conducted by HCI on a scRNA-seq dataset of Diabetes, which successfully clustered the ambiguous cells unassigned in previous study. On the other hand, HCI was also applied to analyze bulk RNA-seq data as previous PFA, e.g., bulk RNA-seq and other omics data (Schuster, 2008). By comparing HCI with the original PFA on three datasets with multiple data types (e.g., gene expression and miRNA expression), it is found that HCI can improve computational efficiency of sample clustering and can recognize gene regulatory networks in an accurate and reliable manner (Joung et al., 2007; Tran et al., 2008; Hamid et al., 2009; Peng et al., 2009).

Totally, HCI can not only cluster cell types with scRNA-seq data in an efficient way, but also capture biologically meaningful sample types as well as extracting network modules with bulk RNA-seq data or other omics data. It provides a new and general way to detect the sample-specific characteristics from the high-order correlation information in an integration manner.

## Materials and Methods

**Figure 1:**
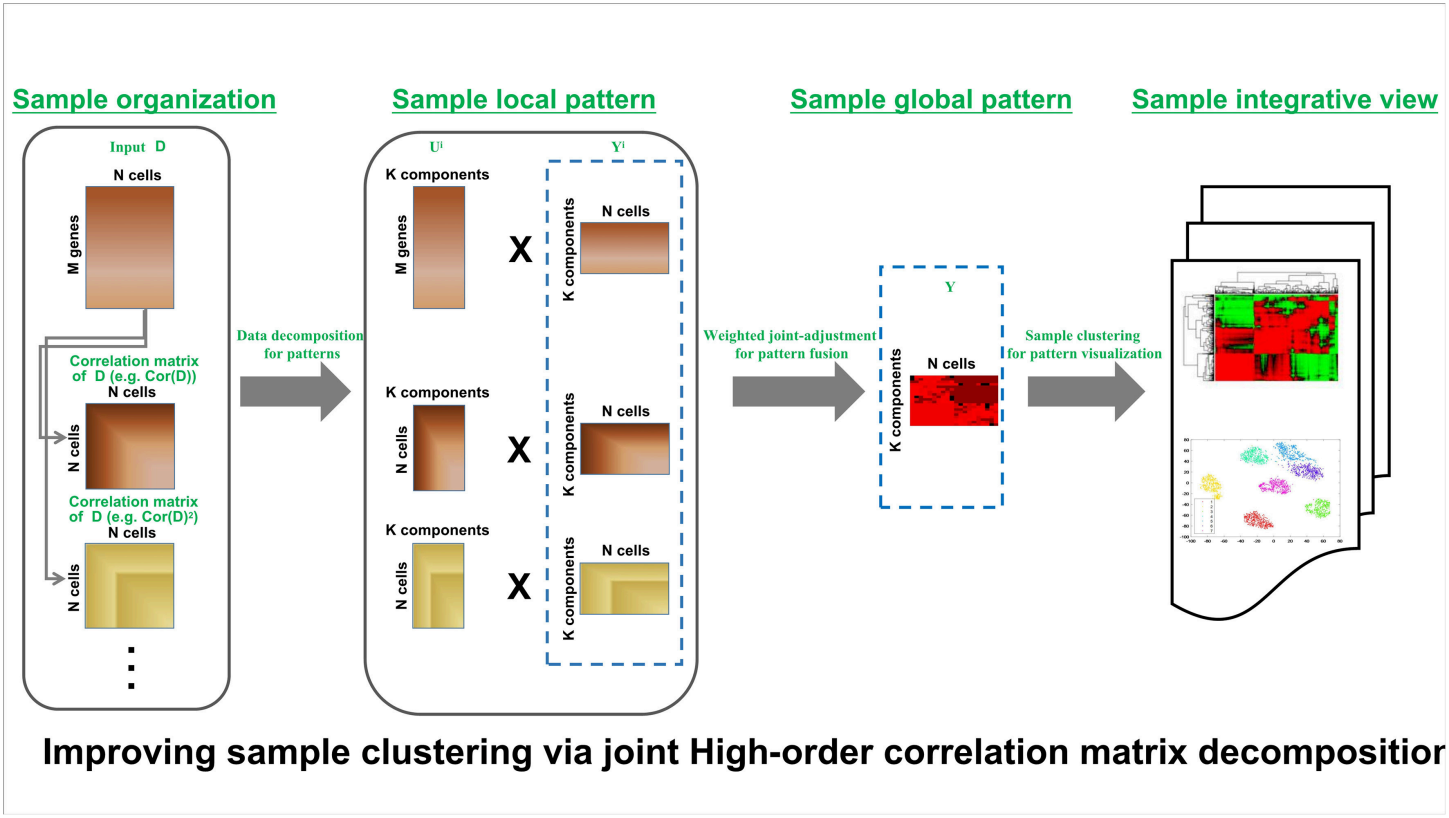
HCI framework of high-order correction matrices based on pattern fusion analysis Overview of single-cell clustering with HCI framework (see section Materials and Methods).

HCI pipeline schematically is shown in Figure 1. Input is the expression matrix **M** where columns correspond to cells or samples and rows correspond to genes or molecules, e.g., each element of **X** corresponds to the expression of a gene in a given cell. The analysis procedure of HCI can be summarized as several steps in follows.

### Pre-processing

The gene filtering removes genes with zero expressions in all cells (or samples), which are not informative for the cell clustering. And, the normalization for each column data is carried to maintain the feature stability of each cell or sample. Then, we can get a filtered expression matrix X.

### High-Order Correlation Matrix Construction

We firstly calculate **F**^1^, the correlation of the gene expression profiles **X**_*m*·*n*_, in which the expressions of *m* genes are measured for *n* samples and *x*_*kj*_ denotes the expression level of gene *k* in sample *j*, the correlation of sample *i* and *j* can be calculated by the Pearson correlation coefficient (Rodgers and Nicewander, 1988):

(1) $$\begin{array}{l} f_{ij}^{\left(1\right)}=\frac{\sum_{k=1}^{n}\left(x_{ki}-x_{-i}\right)\left(x_{kj}-x_{-j}\right)}{\sqrt{\sum_{k=1}^{n}{\left(x_{ki}-x_{-i}\right)}^{2}}\sqrt{\sum_{k=1}^{n}{\left(x_{kj}-x_{-j}\right)}^{2}}} \end{array}$$

where *x*_*ki*_ and *x*_−*i*_ are the expression level of gene *k* and the average gene expression level of sample *i*, respectively. Similarly, *x*_*kj*_ and *x*_−*j*_ are the expression level of gene *k* and the average gene expression level of sample *j*, respectively. Thus, we can obtain a correlation matrix ${\text{F}}_{n\cdot n}^{1}$ of **X** in which $f_{i\cdot j}^{1}$ is its element measuring the correlation coefficient between sample *i* and sample *j*. Now, based on the matrix ${\text{F}}_{n\cdot n}^{1}$, we can further calculate ${\text{F}}_{n\cdot n}^{2}$ as follows:

(2) $$\begin{array}{l} f_{ij}^{\left(2\right)}=\frac{\sum_{k=1}^{n}\left(f_{ki}^{\left(1\right)}-f_{-i}^{\left(1\right)}\right)\left(f_{kj}^{\left(1\right)}-f_{-j}^{\left(1\right)}\right)}{\sqrt{\sum_{k=1}^{n}{\left(f_{ki}^{\left(1\right)}-f_{-i}^{\left(1\right)}\right)}^{2}}\sqrt{\sum_{k=1}^{n}{\left(f_{kj}^{\left(1\right)}-f_{-j}^{\left(1\right)}\right)}^{2}}} \end{array}$$

${\text{F}}_{n\cdot n}^{1}$ is called as the first-order correlation matrix of **X**, and ${\text{F}}_{n\cdot n}^{2}$ is the second-order correlation matrix of **X**. The advantage of this transformation with expression matrix **X** can highlight latent structures between samples with noisy (Hubert, 1985; Ren et al., 2013). In fact, we also investigated the other kind of distance matrix by using other method, such as Spearman correlation, however, ${\text{F}}_{n\cdot n}^{2}$ is similar to ${\text{F}}_{n\cdot n}^{1}$ due to its consideration on element rank rather than element value in matrices. Cleary, the higher-order correlation matrix can be constructed in a similar way. Therefore, in this paper, we only use the Pearson metrics to construct our high-order correlation matrices. Noted, such high-order matrix can enhance the sample clustering performance. In our prior analysis, the clustering accuracy increased quickly on the first-order correlation features, and it almost approached the highest on the second-order correlation features and tended to be saturated when the order further increased. Without loss of generality, we only used the first-order matrix and the second-order matrix to incorporate into HCI in this work.

### Correlation Matrix Induced Pattern Fusion Analysis (PFA)

The input data **X** has *m* rows and *n* columns, and matrices ${\text{F}}_{n\cdot n}^{1}$ and ${\text{F}}_{n\cdot n}^{2}$ have *n* rows and *n* columns. We integrated these three input datasets by pattern fusion analysis. This methodology has been proved and evaluated in previous work (Shi et al., 2017), and the key steps used in our work are as follows:

The first step is to obtain the optimal local information sets of **U**^*i*^, **Y**^*i*^, which requires to minimize the error **E**^*i*^ as follows:

(3) $$\begin{array}{l} min∥E^{i}∥=min_{c^{i},U^{i},Y^{i}}∥W^{i}-\left(c^{i}1^{T}+U^{i}Y^{i}\right){∥}_{F}^{2} \end{array}$$

where **W**^*i*^ is the input data sets **X**, ${\text{F}}_{n\cdot n}^{1}$, ${\text{F}}_{n\cdot n}^{2}$, and *F* is the Frobenius norm. Then, we have

(4) $$\begin{array}{l} \;\{\begin{array}{c} \;U^{i}=Q_{d^{i}}^{i}\\ \;Y^{i}={\left(U^{i}\right)}^{T}\left(W^{i}-c^{i}1^{T}\right)\;\\ c^{i}=\frac{W^{i}1}{n} \end{array} \end{array}$$

where ${\text{Q}}_{d^{i}}^{i}$ is an orthogonal matrix formed by the eigenvectors corresponding to the first *d*^*i*^ largest eigenvalues of (**W**^*i*^ − **c**^*i*^1^*T*^)(**W**^*i*^ − **c**^*i*^1^*T*^)*T*. It is important noted that the sensible default values *d*^*i*^ of matrix *X* is chosen according to $\sum_{r=1}^{d^{i}}\delta_{r}/\sum_{r=1}^{p}\delta_{r}\geq 0.8$ and *d*^*i*^ is the *r* largest eigenvalues of (**W**^*i*^ − **c**^*i*^1^*T*^)(**W**^*i*^ − **c**^*i*^1^*T*^)*T* and the number of the non-zeros eigenvalues is *p*. Meanwhile, the *d*^*i*^-dimension of matrix ${\text{F}}_{n\cdot n}^{1}$ and ${\text{F}}_{n\cdot n}^{2}$ is chosen according to $\sum_{r=1}^{d^{i}}\delta_{r}/\sum_{r=1}^{p}\delta_{r}\geq 0.9$ due to their different feature dimensions with **X**.

And then, the adaptive optimal alignment is used to capture the global sample-pattern matrix **Y**. The detailed adaption method can be seen in the original study (Shi et al., 2017), and the related parameters can be easily adjusted by the user.

### Sample Clustering and Cluster Number Estimation

The global sample-spectrum **Y** obtained in the above step instead of conventional data matrix **X** can be clustered by many clustering methods, such as K-means or HCA. In this paper, K-means clustering (Ding and He, 2004) is performed on the global sample-spectrum matrix *Y* by using the “kmeans()” MATLAB function.

The ratio of distance between clusters (RDC) is calculated to estimate the number *K* of clusters. One hundred realizations of the sample clustering used K-means clustering. The number *K* of clusters is inferred by the average RDC number [*K* = min (*K*, the average RDC's slope is nearly 0)]. The RDC can be calculated as:

(5) $$\begin{array}{l} RDC=\frac{D_{in}}{D_{out}} \end{array}$$

where *D*_*in*_ is the average sample distance in clusters; *D*_*out*_ is the average sample distance between clusters.

Since the reference labels of cells or samples are already known for all published datasets, the Adjusted Rand Index (ARI) (Hubert, 1985) is applied to calculate the similarity between the HCI clustering results and prior-known clusters, which can be further used to evaluate HCI and other methods [e.g., SC3 (Kiselev et al., 2017), PFA, one-order, second-order, and CV situations].

### Molecular Network Construction for Case Study on Bulk RNA-seq Data

The multi-level network is integratively constructed by using HCI schematically shown in **Figure 4A**. In the same way, we calculated the high-order matrices ${\text{F}}_{n\cdot n}^{1}$ and ${\text{F}}_{n\cdot n}^{2}$ of the input datasets **X**_*I*_ (e.g., RNAseq, Methylation, MicroRNA), where *n* is number of samples in data. And then we integrated all input datasets **X**_*I*_ and high-order correlation matrices ${\text{F}}_{I}^{1}$, ${\text{F}}_{I}^{2}$ by using pattern fusion analysis method. Based on the global sample-spectrum matrix **Y**, we can get the differentially expressed mRNAs (or miRNAs) from heterogeneous genomic datasets according to the coefficient matrix **U**^I*^. In this work, we calculated a coefficient of variation for each element on the rows of **U**^I*^:

(6) $$\begin{array}{l} c_{i}=\frac{\delta_{i}}{\mu_{i}} \end{array}$$

where μ_*i*_ is the average weight of mRNA *i* (or miRNA *i*) in *U*^I*^, and δ_*i*_ is the standard deviation. We can define differentially expressed mRNA (or miRNA) *i* if *c*_*i*_ is greater than a given threshold *T*, and they called DEGs (or DE-miRNAs).

Besides, we also performed functional enrichment analysis for genes by Gene Ontology and KEGG. We also analyzed DEGs using Ingenuity Pathway Analysis (IPA), providing the association between a particular gene set and known functions, pathways, networks and associated diseases. An online database miRDB was used for miRNA target prediction and functional annotations.

We defined key genes that significantly enriched in cancer dependent on KEGG, GO and IPA analysis. We found the key genes in the DEGs, which can be linked and correlated by the combined functional couplings of protein-protein interactions of STRING. MicroRNAs which can regulate key DEGs were defined as key miRNAs (degree s > 80) (Hu et al., 2018). Cytoscape was used to reconstruct and visualize gene-gene and miRNA-gene network.

## Results

### Performance Comparison and Robustness Evaluation

**Figure 2:**
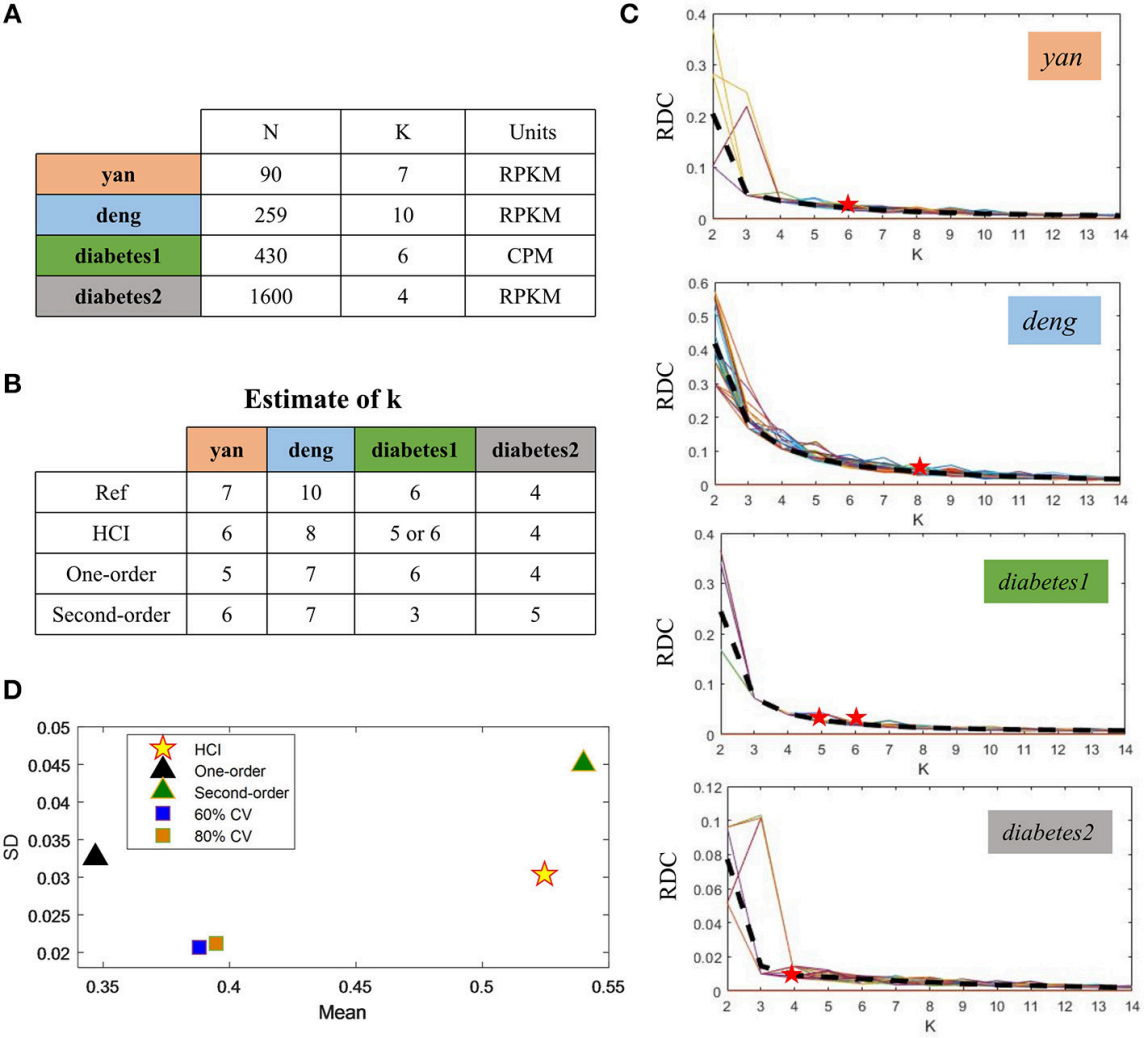
Accuracy and robustness evaluation **(A)** A brief introduction of four published datasets used in HCI. *N* is the number of cells in a dataset; *K* is the number of clusters originally identified; Units: FPKM is Fragments Per Kilobase of transcript per Million mapped reads, CPM is Counts of per Million mapped reads. **(B)** Number of clusters *K* predicted by HCI, One-order situation, Second-order situation, 80% CV used and 60% CV used for all datasets. Ref is the cell cluster reported in previous studies and used as reference of comparison among HCI, One-order situation, and Second-order situation. **(C)** RDC was applied 100 times in global sample-pattern matrix *Y* to each dataset. The solid lines correspond to the value of each RDC calculation. The dashed black lines correspond to the average of these solid lines. Y-coordinate in each graph represents the RDC value and the x-coordinate represents the number of cluster *K*. The star indicates *K* which we choose (see methods). **(D)** The mean and standard deviation of ARI in four datasets by running k-means 50 times separately in different situations.

To demonstrate the performance of HCI on the single-cell datasets, we firstly downloaded four publicly available scRNA-Seq datasets (Figure 2A) (Yan et al., 2013; Deng et al., 2014; Wang et al., 2016; Xin et al., 2016). These datasets were selected on the basis that one can be highly confident on the cell labels as representative cells from different stages, conditions and lines. In order to quantify the similarity between the reference cell types and the clusters obtained by HCI or other comparable methods. We calculated the average ARI of the clustering results (Figure 2D, Figure S1) and estimated cluster number K according to RDC by running K-means 100 times (Figure 2C). Obviously, high-order correlation matrices incorporated into PFA actually improves both the accuracy and the stability of analysis solutions. We found that the accuracy was significantly improved compared with the one-order correlation matrix (only using${\;\text{F}}_{I}^{1}$) or the second-order matrix (only using ${\text{F}}_{I}^{2}$) according to the ARI and the RDC (Figures 2B,**D**). Besides, in order to determine the robustness as a consistent performance under different conditions, the same analysis on four datasets were both repeated 50 times under different systematic conditions (e.g., 60% CV genes or 80% CV genes used) respectively, where CV genes mean ones with largest expression variances. Similarly, the performance of HCI under different correlation matrices or conditions was better (i.e., robust) than other methods according to the ARI and the RDC (Figures 2B,D, Figure S1). Overall, HCI always outperformed compared methods on distinguishing single-cell types.

### Comparison of Sample-Cluster Identification With One-Level Data

We applied HCI and SC3 method to the above four datasets for evaluation and comparison on the cell clustering. We calculated the cluster number K and the running time in each individual dataset by using the R package of SC3 (Kiselev et al., 2017). On the one hand, as shown in Table 1, HCI performs better than SC3 across almost all datasets in estimating the number K of clusters (except for similar performance on Deng dataset). On the other hand, the running time of 2,000 cells for SC3 is more than 1 h. By contrast, the running time of HCI for 2,000 cells is <10 min as shown in Table 2. It is worth noted that HCI can even apply to large datasets, such as: 10k datasets from 10x genomics, with more than 10,000 cells by using MATLAB efficiently (Table 2, Figure S2). From these results, we included that, HCI has better performance than SC3 because it considers the high-order correlation information, and integrates this potential heterogeneous information by our PFA framework well.

**Table 1:** The estimation of K compared with SC3 on real datasets.

	**yan**	**deng**	**diabetes1**	**diabetes2**
Ref	7	10	6	4
cPFA	6	7	5 or 6	4
SC3	6	9	11	13

**Table 2:** The running time compared with SC3 on real datasets.

	**yan**	**deng**	**diabetes1**	**diabetes2**	**brain**	**10x**
N cells	90	259	430	1,600	3,003	10,000
cPFA	5 s	18 s	31.85 s	5.8 min	16.4 min	3.1 h
SC3	7.33 min	18 min	29.67 min	101.18 min	98 min	4 h (no result)

### Case Study on the scRNA-seq Data of Diabetes

**Figure 3:**
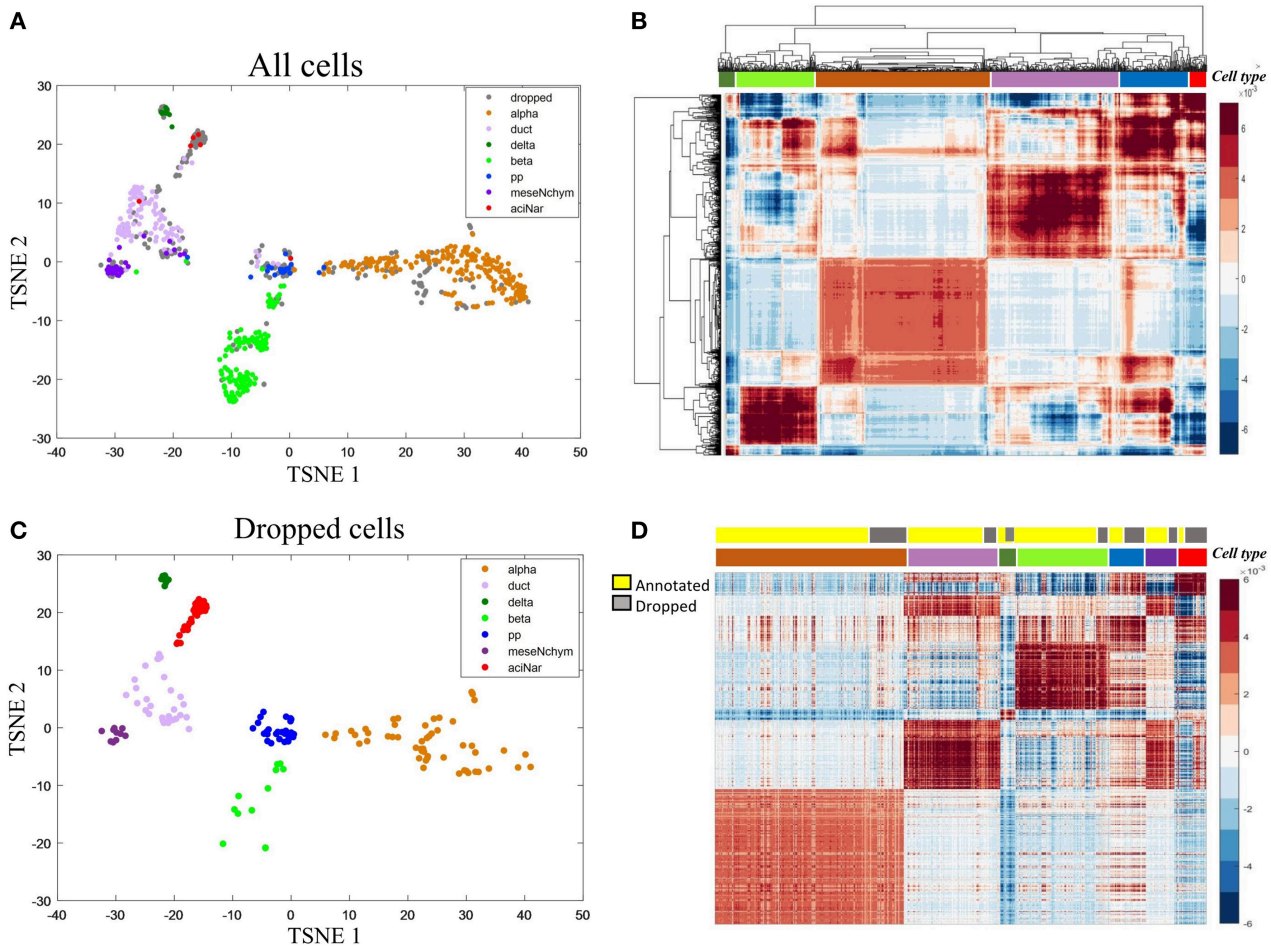
Case study on the diabetes **(A)** The two-dimensional projection of the global-sample pattern matrix *Y* of diabetes1 dataset using t-SNE. Colors represent different cell types where gray dots specifically mean dropped cells. **(B)** Hierarchical clustering diagram of all cells. The colors at the top represent the references labels (see color legend in **A**). The purple represents the ductal cell and mesenchymal cell because these two cells are mixed together. **(C)** For the previously dropped cells, HCI can cluster them into different groups corresponding to known cell types well. Thus, it is more efficient on cell type identification with less non-identified cells. **(D)** Heatmap representation of *Y* of all cells. The first color bar represents the type of annotated cell and ambiguous cells and the second bar represents the seven cell types as the same as in **(A)** legend.

We then applied HCI to a diabetes scRNA-seq data (Wang et al., 2016) with 430 annotated cells belonging to six cell types, where 205 ambiguous cells previously unassigned. For the 430 annotated cells, the RDC of HCI suggested that K is 5 or 6 (Figures 2B,**D**), provides the reasonable cluster number of cells. When we applied HCI to the whole cells included 430 annotated cells and 205 dropped cells, the results suggested that the K is 7. Obviously, there are potential new cell types included, and we found there were 27 annotated mesenchymal cells in the ambiguous cells. This result also showed that the other ambiguous cells can be clustered well in seven cell types separately (Figure 3A). Besides, the other methods (e.g., tSNE, HCA) were used to visualize the clusters of these dropped cells (Figure 3). As a control to this analysis, one well-known scRNA-seq analysis method SEURAT (Macosko et al., 2015) was also applied. As the results shown (Figure S3), HCI performed better than these traditional methods on distinguishing cell types. Noted, cluster dendrogram of global sample-pattern matrices **Y**, **F**^1^, and **F**^2^ are shown in Figure S4 for illustrating the influence of HCI on information integration.

In addition, marker genes are particularly useful since they can usually uniquely indicate a cell cluster, e.g., α-cells with high expression on *IRX2* and *ARX*. To further interpret the biological meaning of HCI based cell clustering, we applied the 50 key marker genes of the annotated cell types to categorize the previously dropped cells which had been clustered well by HCI now. The violin plot shown the expression level of *IRX2* and *ARX* are significantly high in alpha cells previously identified and also in alpha-dropped cells newly clustered by HCI (Figure S5). Furthermore, it was observed a high degree of expression similarity between annotated cells and their corresponding clustered-dropped cells in these key markers (Figures S6, S7). Together with these results, we concluded that HCI is able to identify new cell types with high accuracy and biological significance.

### Comparison of Sample-Cluster Identification With Multi-Level Data

**Figure 4:**
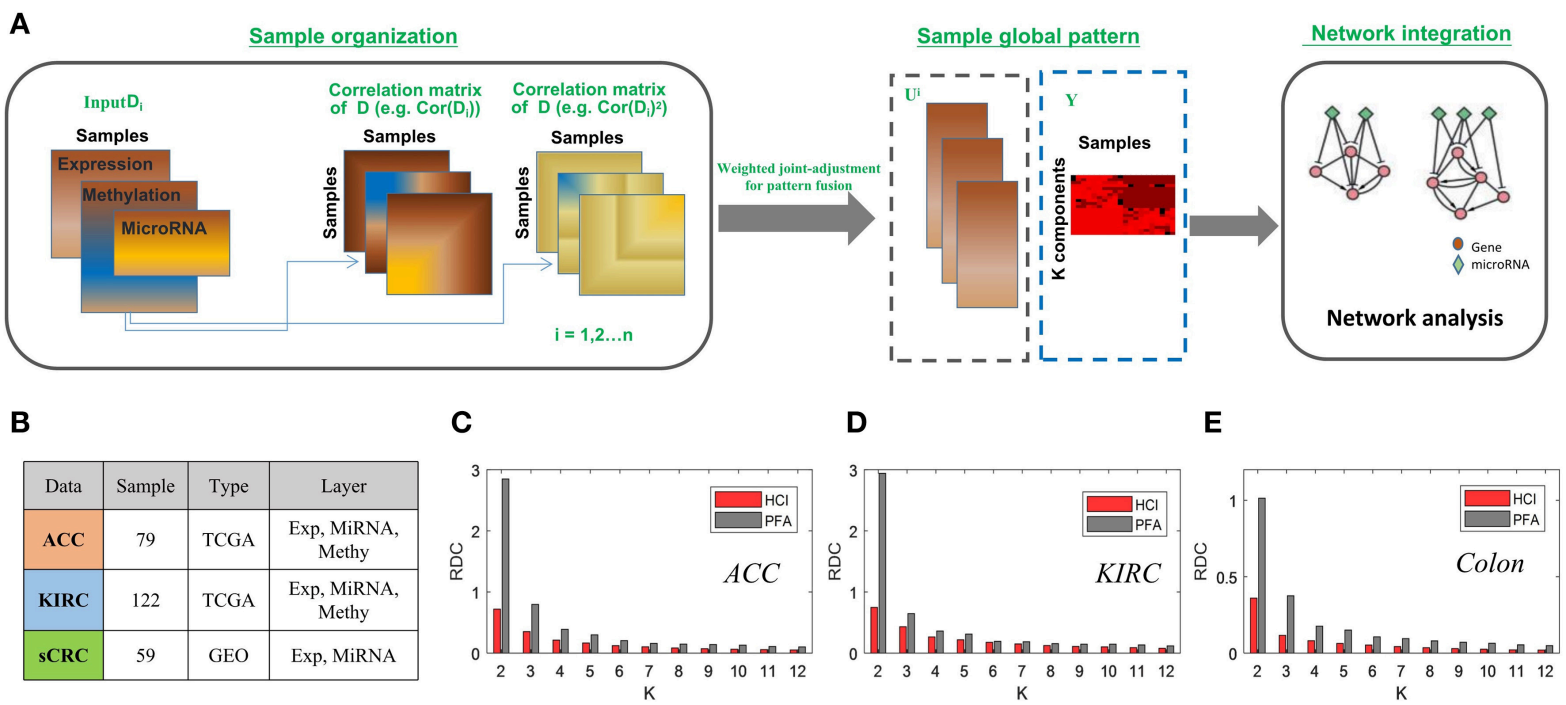
The enhanced framework flow for integrating bulk datasets and comparison of PFA **(A)** The flow chart of HCI to integrating multiple heterogeneous omics data **(B)** A brief introduction to the datasets we used in this comparison. **(C–E)** Bars correspond to the average of the RDC values by running 100 times in each dataset. Red and gray colors correspond to result of HCI and PFA respectively.

To demonstrate the effectiveness of HCI inherited from PFA for integrating multi-level datasets, we applied HCI to three cancer omics datasets, two from the TCGA Data Portal included kidney renal clear cell carcinoma (KIRC) and Adrenocortical carcinoma (ACC), and one from the GEO (Colorectal cancer) (Sayagués et al., 2016). For the two TCGA data, the gene expression, miRNA expression and DNA methylation profiles were prepared in a similar way as those in Shi et al. (2017). As for the Colon cancer, the gene expression and miRNA expression were obtained, and we removed those mRNAs or miRNAs if they have more than 80% zero expression values across all samples. Then these datasets with 122 patients in KIRC, 79 in ACC and 51 in colon cancer were prepared, respectively (Figure 4B).

After carried on HCI and PFA on these datasets, respectively, we compared their results according to the RDC, which show that that HCI indeed performs better in terms of accuracy of cluster quality across datasets (Figures 4C–E). In this comparison, the heterogeneity factors including different complex conditions, varying data resources and dissimilar samples size would provide strong evidences to support the ability of HCI on identifying clinically relevant disease subtypes and predicting network modules involved in complex diseases (Zhang et al., 2011; Zang et al., 2016).

### Case Study on the Matched mRNA and miRNA Data of Colorectal Cancer

**Figure 5:**
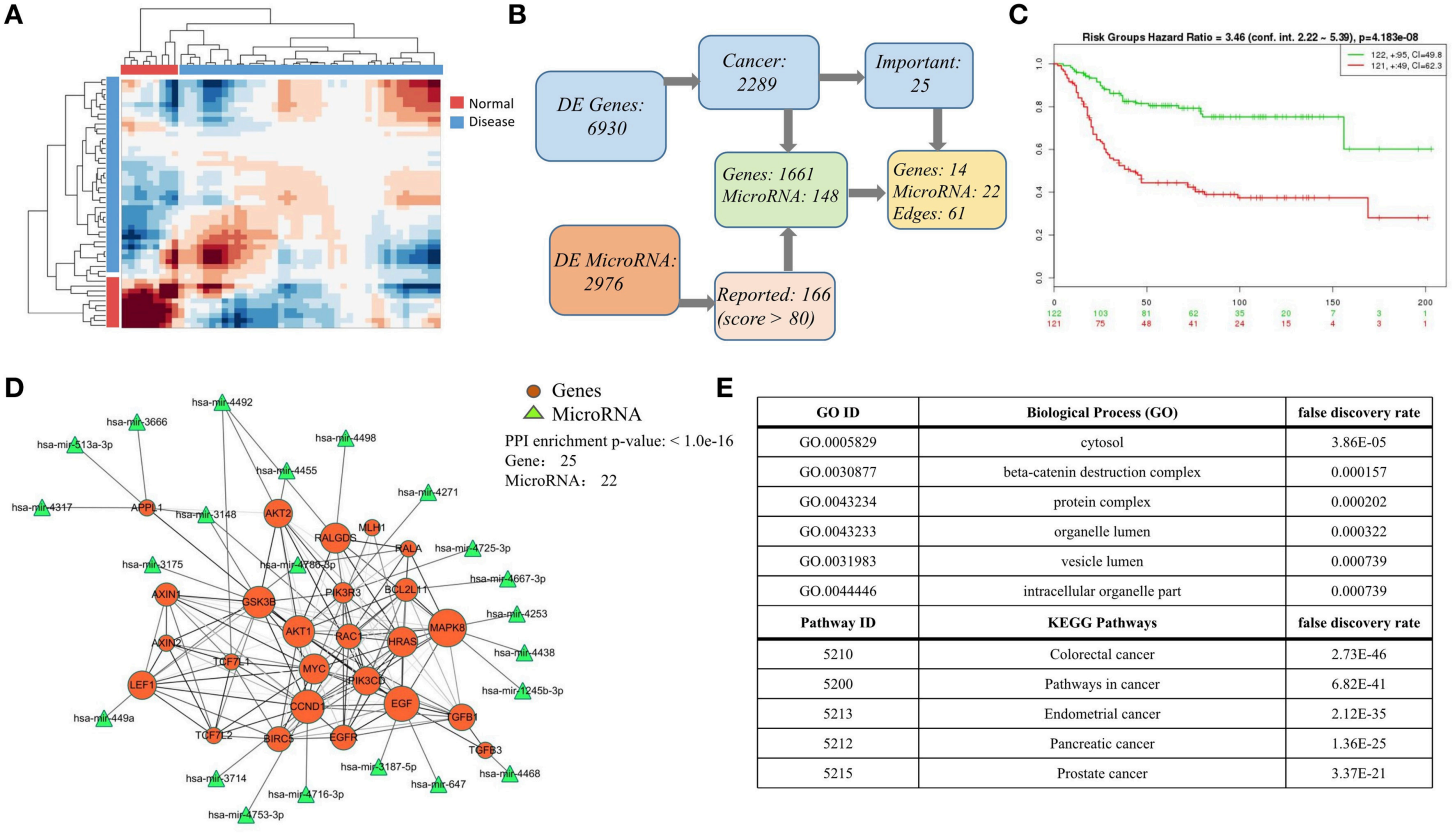
Case study on the colorect cancer **(A)** Hierarchical clustering diagram of samples in matrix *Y*. Color bars represent the normal samples and disease samples. **(B)** The process diagram of selecting key genes. **(C)** Evaluation of the selected 25 important genes related to colorectal cancer in **(B)**. In SurvExpress, we used the average for selected genes, two risk groups and Cox fitting to generate Survival curves. The total number of each group is shown in the top right corner of graph, and the number of censoring samples is marked with +. The CI per curve is also included. *P*-value is shown in the top of figure. **(D)** The highly connected network consists mainly of 25 DEG genes and 22 miRNAs. The 22 miRNAs targets 16 genes based on miRDB database. The size of node indicates the network degree of gene. And the PPI enrichment *P*-value of genes is shown in the top right corner of this figure. **(E)** Top-ranked pathways and biological functions enriched in the 25 genes in **(D)**.

Finally, we carried on a case study again on colorectal cancer data, especially providing the integrated mRNA-miRNA network according to the global sample-spectrum matrix **Y**. Firstly, the HCI results suggested that the normal (9 samples) and disease (42 samples) can be clustered into two discriminative groups (Figure 5A). Then, 6,930 differentially expressed genes and 2,976 differentially expressed miRNAs were obtained. By functional enrichment analysis on these differentially expressed genes with GO BP terms, KEGG pathways and IPA annotations, all significant physiological system development, function terms, disease and networks are listed in Tables S1, S2. We found that there are 2,289 genes (nearly 33% DEGs) are significantly correlated with colon cancer among all DEGs. Besides, according to the miRNA target predication from miRDB, 1,661 DEGs can be regulated by 141 DE-miRNAs (Figure 5B). Note that all enrichment analysis results involve 25 key genes, 14 of which can be regulated by 22 key miRNAs (Figure 5B). In addition, the survival risks of these genes were also evaluated as shown in Figure 5C.

As an illustrative instance, we constructed the gene-gene network of 25 key genes (Figure 5D) based on the STRING (*p* = 1.0e-16) (2018)^2^. The enrichment analysis results of this network are listed in Figure 5E (Table S3), and this network is significantly enriched with cytosol (*P* = 3.86e-05), beta-catenin destruction complex (*P* = 1.57e-04), colorectal cancer (*P* = 2.73e-46), and pathways in cancer (*P* = 6.82e-41). We also found that the hub genes (e.g., *MAPK8, EGF, FALGDS, CCND1, MYC*) in this network have been linked to cancer in wide literature reports. For example, the MAPK-signaling pathways have been identified as one of the most strongly associated gene markers to colorectal cancer (CRC) (Cummins et al., 2006; Barault et al., 2008; Lascorz et al., 2010; Slattery et al., 2012). *MAPK8* has been shown to interact with *MYC* which is frequently observed in numerous human cancers. Strikingly, 22 key miRNAs are correlated with 14 key genes in this network. *MiRNA-647* and *miRNA-449a* have been reported their association with colorectal cancer (Noguchi et al., 1999; Feng et al., 2018). These results revealed HCI would classify the sample types clearly and could integrate the multi-level regulatory network based on multiple heterogeneous data. All relevant DEGs and DE-miRNAs are worthy of future experimental investigation, and listed in Tables S4, S5.

## Discussion and Conclusion

The distinct types of biological data could provide a precise explanation for understanding the complex biological processes (Ghazalpour et al., 2006; Kutalik et al., 2008; Li et al., 2012; Zhang et al., 2012; Chen and Zhang, 2016; Zeng et al., 2016; Feng et al., 2018; Yu and Zeng, 2018). In recent decades, many approaches were proposed for analyzing single-cell data or multi-omics data to identify subtypes and construct biological networks (Gygi et al., 1999; Ding and He, 2004; Chari et al., 2010; Zhang et al., 2011; Kiselev et al., 2017; Guo et al., 2018a,b; Wang et al., 2018). However, for most methods, there are some limitations on reliably identifying the sample types by exploiting multi-datasets, such as the effect of noise on data and the computational cost. And some methods would fail to make full use of the similarity information between samples, thus making the results unreliable. Hence, in order to overcome this problem, a flexible and efficient integration method with automated information fusion and bias correction is demanded. In this work, we introduced the data-driven integrating method HCI. The key idea of this method is to incorporate the high-order similarity matrices (e.g., Pearson correlation matrix) into pattern fusion analysis, where the sample cluster or subtype structure can be actually determined benefiting from the high-order correlations. And the obtained combinatorial sample patterns from HCI could represent comprehensive characterization of inherent sample relations in data. In order to demonstrate the benefits of HCI, various evaluations have been carried on both scRNA-seq and bulk RNA-seq datasets for complex diseases. As expected, HCI effectively captured the sample (e.g., cell or patient) clusters and outperformed the existing methods under different conditions in terms of accuracy and robustness. And two deep case studies supported that HCI has satisfactory flexibility and applicability. Noted, HCI is based on PFA, which has been evaluated and compared with a few multi-view clustering methods in previous study (Shi et al., 2017). Meantime, SC3 has also been evaluated and compared with many existing approaches (Kiselev et al., 2017). Thus, in this study of scRNA data, we have directly compared HCI and SC3 on multiple datasets. It is worthy to carry on more benchmark studies in this field as a future topic (Zeng et al., 2016). Also as a future topic, we can improve HCI by further exploiting dynamics and network information, such as applying network biomarker (Zhang et al., 2015; Liu et al., 2016; Zhao et al., 2016; Liu, X. et al., 2018) or applying dynamic network biomarker (Chen et al., 2012; Li et al., 2017; Liu et al., 2017; Liu, R. et al., 2018; Yang B. et al., 2018) for accurate and reliable clustering and classification based on omics data from the perspectives of dynamics and network.

As genomic data sources is increasing in diversity and volume, HCI can fit the data structures on both one level data or multiple level data, so that, HCI could provide new avenues for the systematic explanation of various data and complex biological phenotypes at a system-wide level. Indeed, there are still a few future topics to further extend HCI method, e.g., integrating discrete data types including somatic, SNP, and CNV information.

## Author Contributions

HT and TZ developed the methodology. HT executed the experiment. HT and TZ carried out the data analysis and wrote this paper. HT, TZ, and LC revised the manuscript. LC and TZ supervised the work, and LC critically reviewed the paper. All authors read and approved the final manuscript.

### Conflict of Interest Statement

The authors declare that the research was conducted in the absence of any commercial or financial relationships that could be construed as a potential conflict of interest.

## Footnotes

^1^10X Genomics single cell gene expression datasets from https://support.10xgenomics.com/single-cell-gene-expression/datasets

^2^https://string-db.org/
