# Supplementary material for: High-Order Correlation Integration for Single-Cell or Bulk RNA-seq Data Analysis
Source: Front Genet. 2019 Apr 26;10:371. doi: 10.3389/fgene.2019.00371 (PMC6497731; doi:10.3389/fgene.2019.00371)
Supplement: Supplementary file 1 [file Table_1.docx]

**Supplementary of High-order correlation integration for single-cell or bulk RNA-seq data analysis**


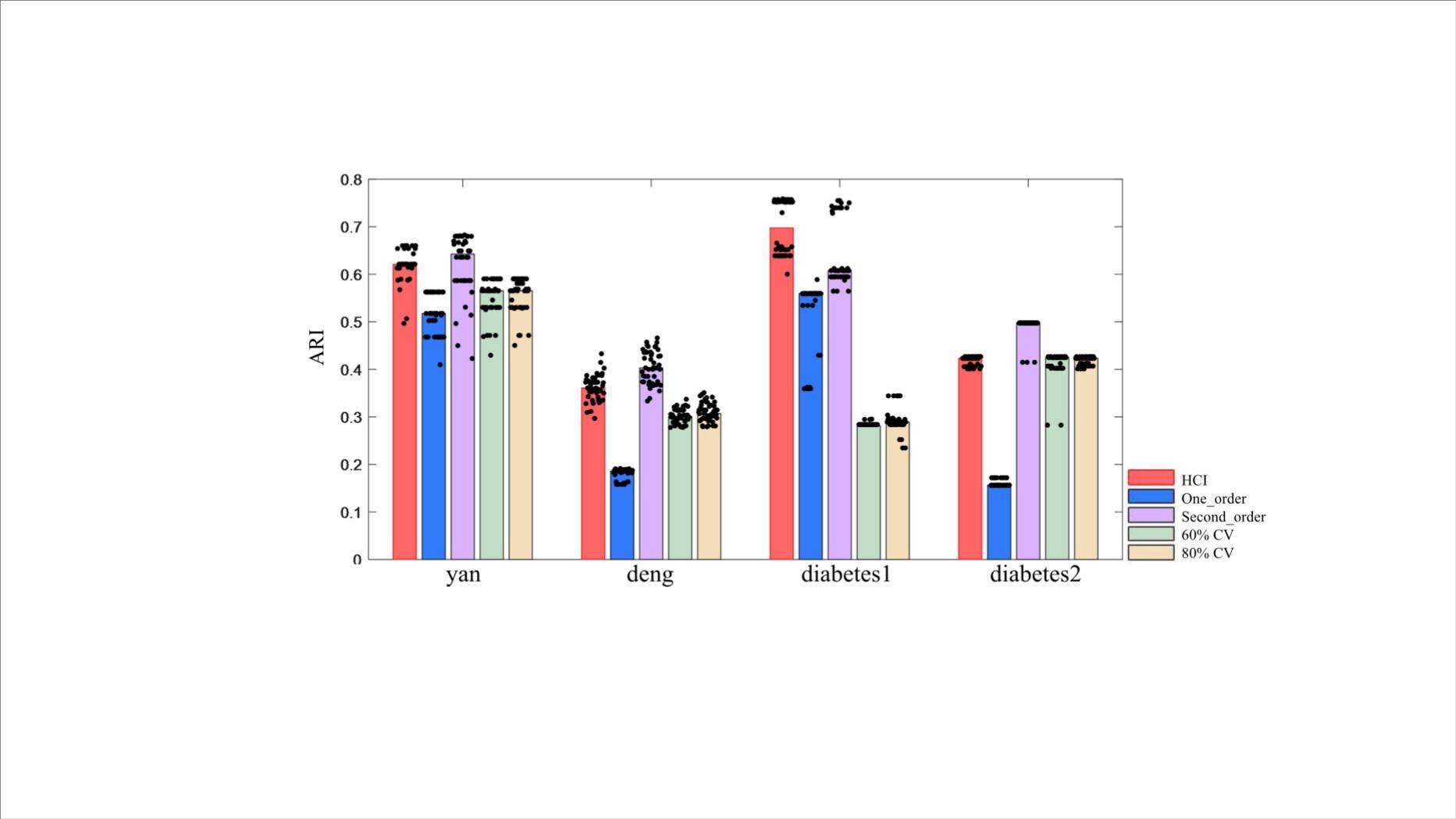


Figure S1 Analysis of HCI, one-order correlation matrix, second-order correlation matrix. 60% CV, 80% CV were applied 50 times to each global sample-pattern matrix $\mathbf{Y}$. Each panel shows the ARI (black dots, Methods) between the inferred clusters and the reference labels. Bars correspond to the median of the dots.


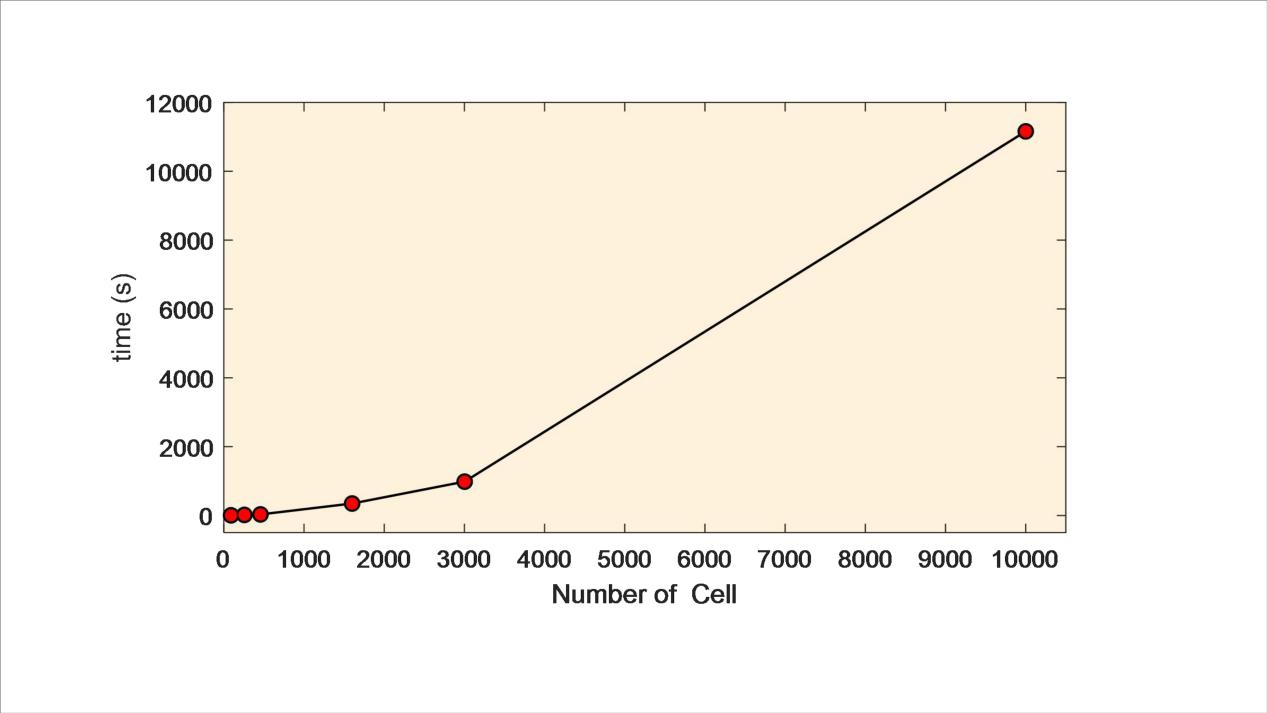


Figure S2 Run times for different number of cells. Data sets are yan, deng, Diabetes1 Diabetes2, GSE60369 and 10K cells from a MALT Tumor downloaded in 10x genomics.


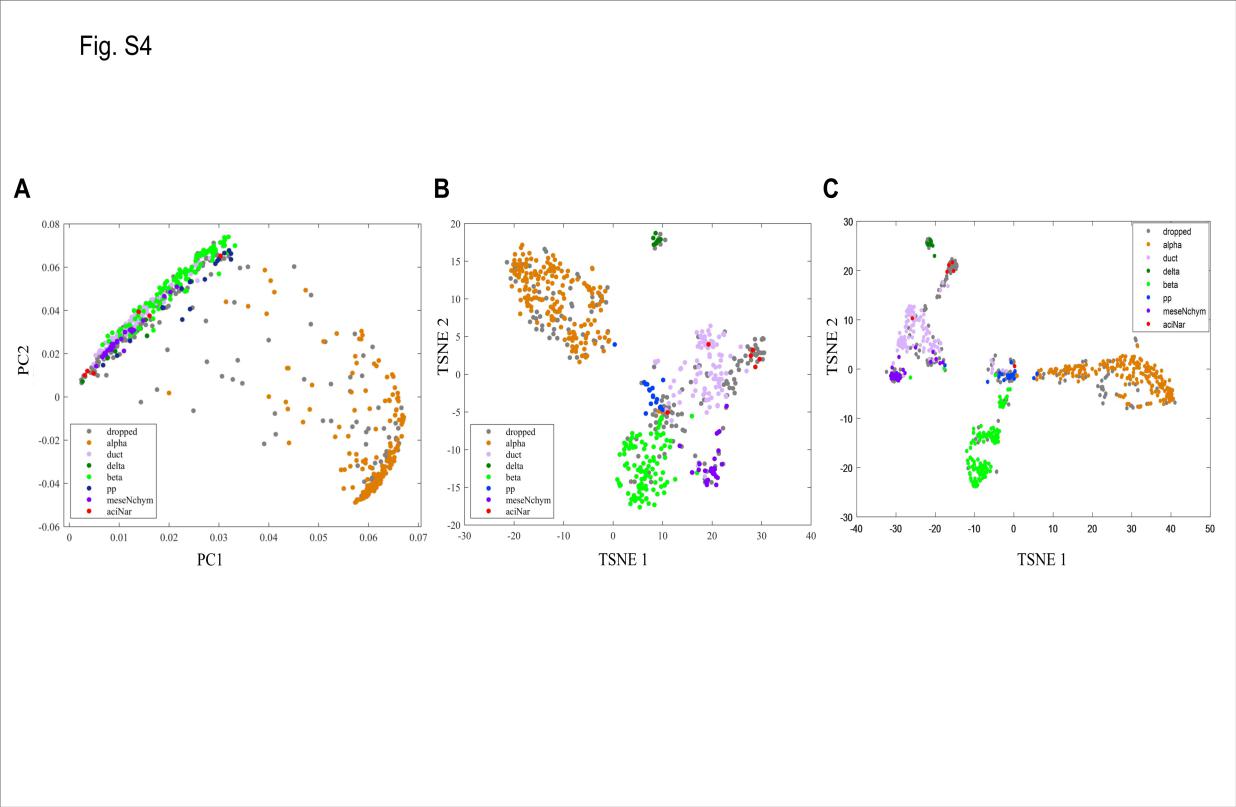


Figure S3 the clustering results of PCA, TSNE and HCI. (A) Principle component analysis by using SEURAT. (B) The two-dimensional projection of diabetes data using TSNE. (C) The two-dimensional projection of the global-sample pattern matrix using t-SNE. Colors represent different cell types.


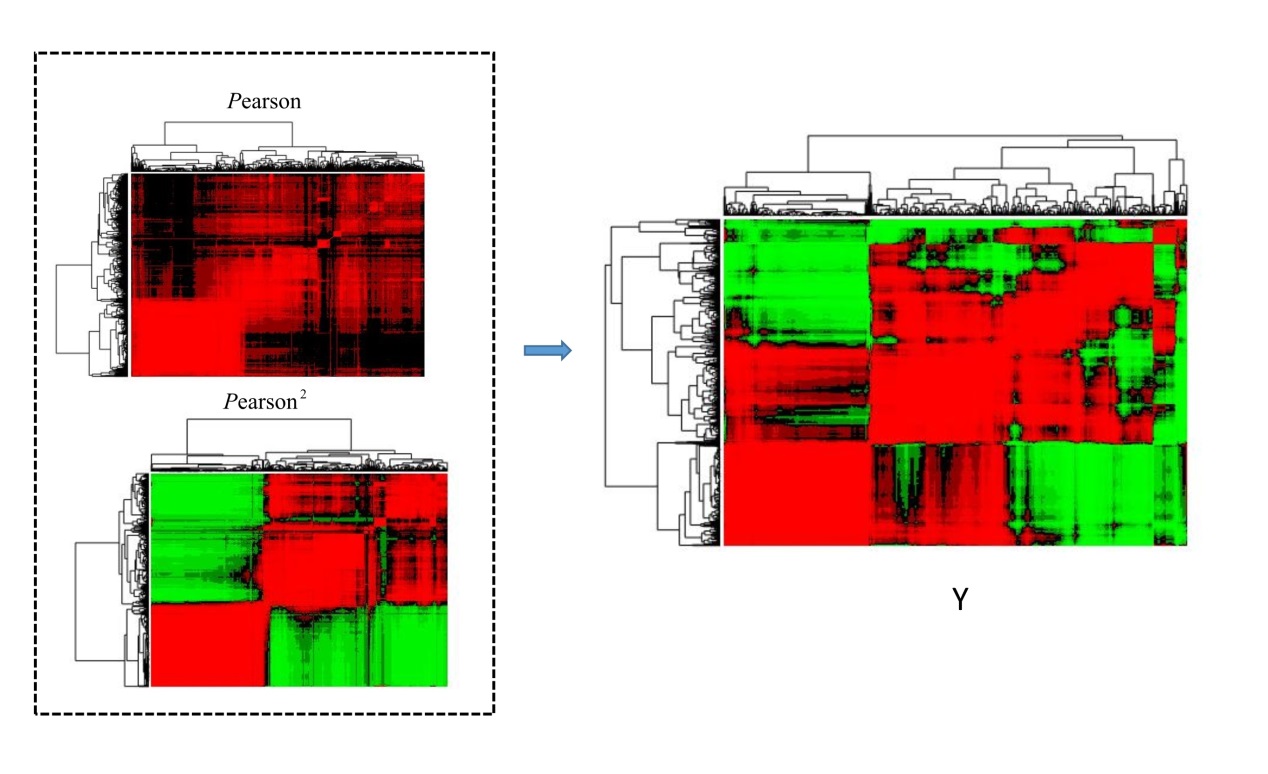


Figure S4 Cluster dendrogram of global sample-pattern matrices $\mathbf{F}^{\boldsymbol{1}}$, $\mathbf{F}^{\boldsymbol{2}}$ **and** $\mathbf{Y}$


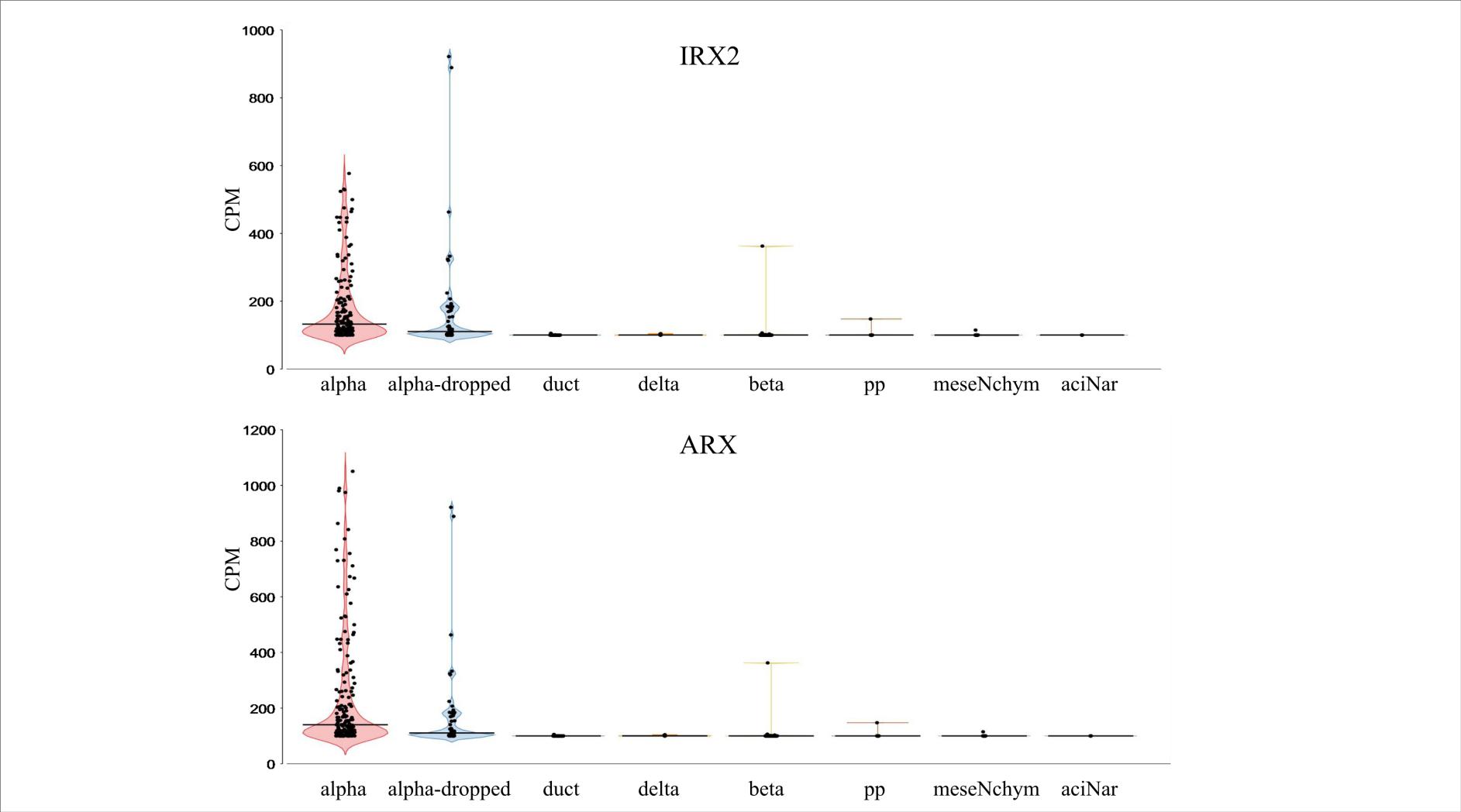


Figure S5 Violin plots of the expression values of selected markers in alpha cells, alpha-dropped cells clustered by HCI and other cell types. *IRX2* and *ARX* have high expression in alpha cells and alpha-dropped cells.


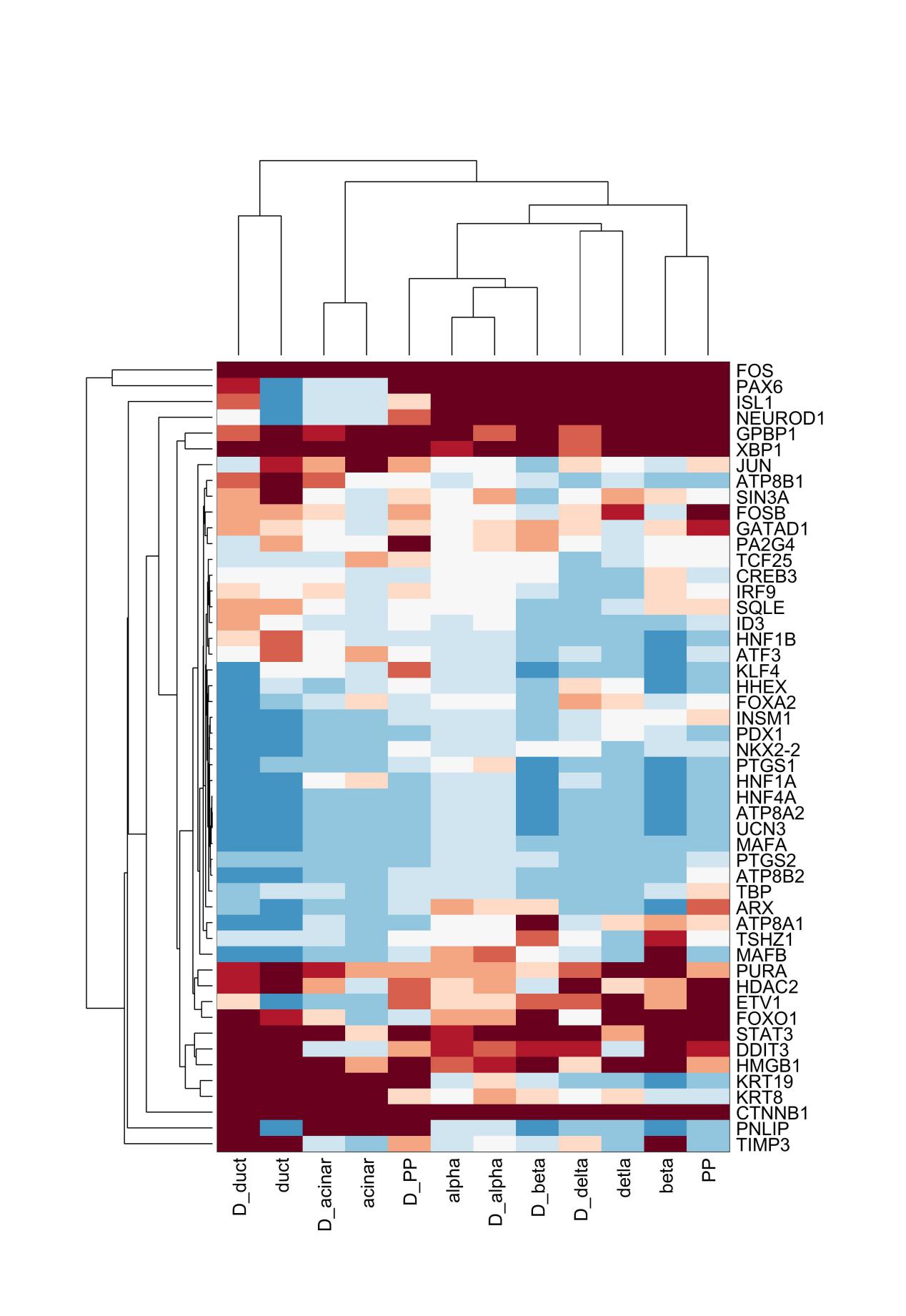


Figure S6 Average expression of key markers in the six annotated cell types and their corresponding clustered-dropped cell types by using HCI. Samples and genes are clustered by Pearson correlation distance.

Figure S7 the correlation of the average expression between annotated cell types and their corresponding clustered-dropped cell types. Bars correspond to the value of correlation. The dotted red line corresponds to correlation=0.6.

Table S1 The list of GO biological process terms and KEGG pathways. The details are given in an additional file named as EnrichmentResult.xlsx.

Table S2 The IPA analysis result. The details are given in an additional file named as IPAresult.xlsx.

Table S3 The PPI enrichment result of 25 genes.

| pathway ID | Biological Process (GO) | false discovery rate |
| --- | --- | --- |
| GO.0005829 | cytosol | 3.86E-05 |
| GO.0030877 | beta-catenin destruction complex | 0.000157 |
| GO.0043234 | protein complex | 0.000202 |
| GO.0043233 | organelle lumen | 0.000322 |
| GO.0031983 | vesicle lumen | 0.000739 |
| GO.0044446 | intracellular organelle part | 0.000739 |
| pathway ID | Molecular Function (GO) | false discovery rate |
| GO.0008013 | beta-catenin binding | 1.58E-07 |
| GO.0019899 | enzyme binding | 5.85E-07 |
| GO.0070016 | armadillo repeat domain binding | 5.85E-07 |
| GO.0005515 | protein binding | 5.76E-06 |
| GO.0030234 | enzyme regulator activity | 2.00E-05 |
| pathway ID | KEGG Pathways | false discovery rate |
| 5210 | Colorectal cancer | 2.73E-46 |
| 5200 | Pathways in cancer | 6.82E-41 |
| 5213 | Endometrial cancer | 2.12E-35 |
| 5212 | Pancreatic cancer | 1.36E-25 |
| 5215 | Prostate cancer | 3.37E-21 |
| pathway ID | Cellular Component (GO) | false discovery rate |
| GO.0007167 | enzyme linked receptor protein signaling pathway | 6.40E-16 |
| GO.0071310 | cellular response to organic substance | 4.56E-14 |
| GO.0010033 | response to organic substance | 2.16E-13 |
| GO.0007169 | transmembrane receptor protein tyrosine kinase signaling pathway | 2.38E-13 |
| GO.0071363 | cellular response to growth factor stimulus | 2.44E-12 |

Table S4 Differential expressed genes are list in an additional file called DEGs.xlsx.

Table S5 Differential expressed miRNAs are list in an additional file called DEmiRNAs.xlsx.
